# Supplementary material for: FindZebra online search delving into rare disease case reports using natural language processing
Source: PLOS Digit Health. 2023 Jun 29;2(6):e0000269. doi: 10.1371/journal.pdig.0000269 (PMC10309602; doi:10.1371/journal.pdig.0000269)
Supplement: S1 Text — (DOCX) [file pdig.0000269.s001.docx]

# Supporting Information

## Collection of PubMed case report abstracts

Abstracts were downloaded from the PubMed database using the ESearch utility which is part of the [Entrez Programming Utilities](https://www.ncbi.nlm.nih.gov/books/NBK25501/). The following command structure was used for each disease to generate the extracts and save them in XML format:

./esearch -db pubmed -query "<<PubMed search query>>" | ./efetch -format xml > <<output file>>

The search queries themselves consist of the most prominent name or names of each disease, a clause to narrow the search to focus on case reports, and a filter to only get results that have an abstract available to be included in the abstract: “<<disease name or names>> and (case report or case reports) has abstract [FILT]”. A few abstracts are removed after retrieval because they turn out to be empty. For Gaucher and Fabry we retrieve 803 and 883 abstracts, respectively. The S1 Table gives the details for the two diseases.

| S1 Table. ESearch queries | | | |
| --- | --- | --- | --- |
| **Disease** | **Search query** | **Latest retrieval** | **# results** |
| Gaucher’s disease | gaucher disease and (case report or case reports) has abstract [FILT] | 2022-03-03 11:38 CET | 803 |
| Fabry disease | fabry disease and (case report or case reports) has abstract [FILT] | 2022-03-03 11:45 CET | 883 |

We have deliberately chosen to 1) exclude the full text of the articles and 2) restrict retrieval to case reports. Full-text retrieval is possible for open-access articles but requires adapting the retrieval to individual publishers. To make a robust solution is time-consuming to establish and maintain and we have reasoned that the key information needed for finding the phenotype is present in the abstract. A wider search query will retrieve case reports not tagged as such but will also retrieve less relevant abstracts (reviews, etc).

## Takeda-owned Outcomes Surveys data

The Fabry Outcome Survey (FOS) is a prospective, multicenter, observational, open-ended disease registry designed to document the clinical outcome over time of patients with Fabry disease, irrespective of the treatment status. Each patient to be enrolled in this registry must have a documented confirmed diagnosis of Fabry disease. There is no predetermined sample size. Participation in FOS is voluntary (at the discretion of the investigator and the patient) and no treatment will be provided to the patient as a result of participating in this study. All treatments and care of patients and/or any changes considered necessary for a patient’s welfare will be determined at the discretion of patients’ physicians, irrespective of participation. Patients will be followed in the registry for as long as the investigator and patient deem appropriate. Comprehensive data from routine patient visits and assessments for the management of Fabry disease is entered into the registry database at baseline and after follow-up visits. FOS will provide the opportunity to collect and report clinical outcome assessments designed to increase the understanding of the course of Fabry disease, irrespective of patients’ treatment. Data collected in the FOS registry may also provide information to healthcare professionals about disease treatment options. Patients included may be untreated or currently or previously treated with agalsidase alfa or any other approved treatment for Fabry disease.

The Gaucher Outcome Survey (GOS) is a long-term observational survey being developed in conjunction with leading international experts on Gaucher disease. Patient participation in GOS is voluntary and GOS is open to patients of any age or gender with a confirmed diagnosis (biochemical and/or genetic) of Gaucher disease of any phenotype. Patients who are naïve to treatment, patients who are currently or have been previously treated with velaglucerase alfa, as well as patients who have been exposed to or are currently receiving other treatments for Gaucher disease are included. It is designed to gain a better understanding of the clinical course of the disease and its response to velaglucerase alfa therapy, thereby improving the clinical management of patients affected by Gaucher disease. Clinical and laboratory tests that are part of standard medical care for patients with Gaucher disease are performed for all patients and their results are recorded and entered into GOS. The collection of additional information is possible as part of specific sub-studies to answer questions of clinical importance and to potentially aid in the management of patients with Gaucher disease.

## Text Segmentation

The segmentation model is used on FindZebra.com to 1) parse the queries, 2) index the PubMed case reports and 3) improve the readability of the returned abstracts. In the following sections, we detail the fine-tuning process and display segmentation samples for three documents.

### Fine-tuning

The hyperparameters were optimised to maximise the F1 score with cross-validation. The F1 score is computed at the word level (such as to be independent of the choice of tokenizer) using the SpaCy library. We searched across numerous architectures, including different medical BERT models (BioBERT, SciBERT, ClinicalBERT, and BioMed-RoBERTa), different output distributions (categorical independent, autoregressive left-to-right, CRF), different parameterizations of the output distribution (BERT + multilayer-perceptron, BERT + LSTM), different levels of regularisation (dropout), and different span encoding schemes (no encoding, BIO, BILOU). The final model uses PubMedBERT with one projection layer, a BILOU encoding scheme, and a dropout rate of 0·3 for the attention mechanism, and 0·1 for the rest.

### Segmentation samples

In this section, we showcase two examples of segmented PubMed abstracts that were not included in the training set (Gaucher; S1 Fig and Fabry; S2 Fig). We showcase one article for Fabry and one for Gaucher. To test the robustness of the model to documents that are out of domain (not Fabry and not Gaucher), we segmented a case report for another disease (COVID-19; S3 Fig).

#### S1 Fig. Gaucher: “Rare GBA1 genotype associated with severe bone disease in Gaucher disease type 1”


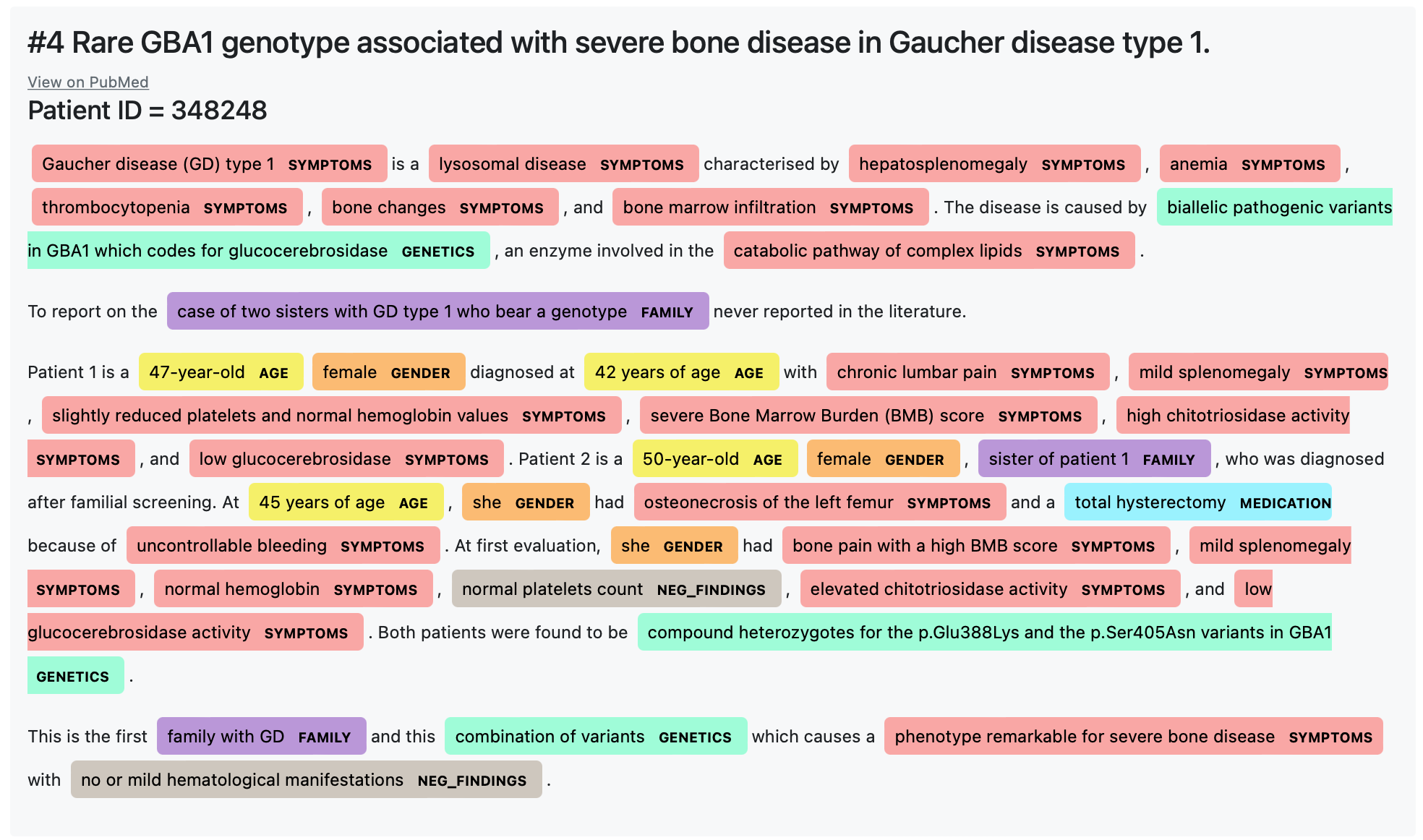


#### S2 Fig. Fabry: “Significant improvement in Fabry disease podocytopathy after 3 years of treatment with agalsidase beta”


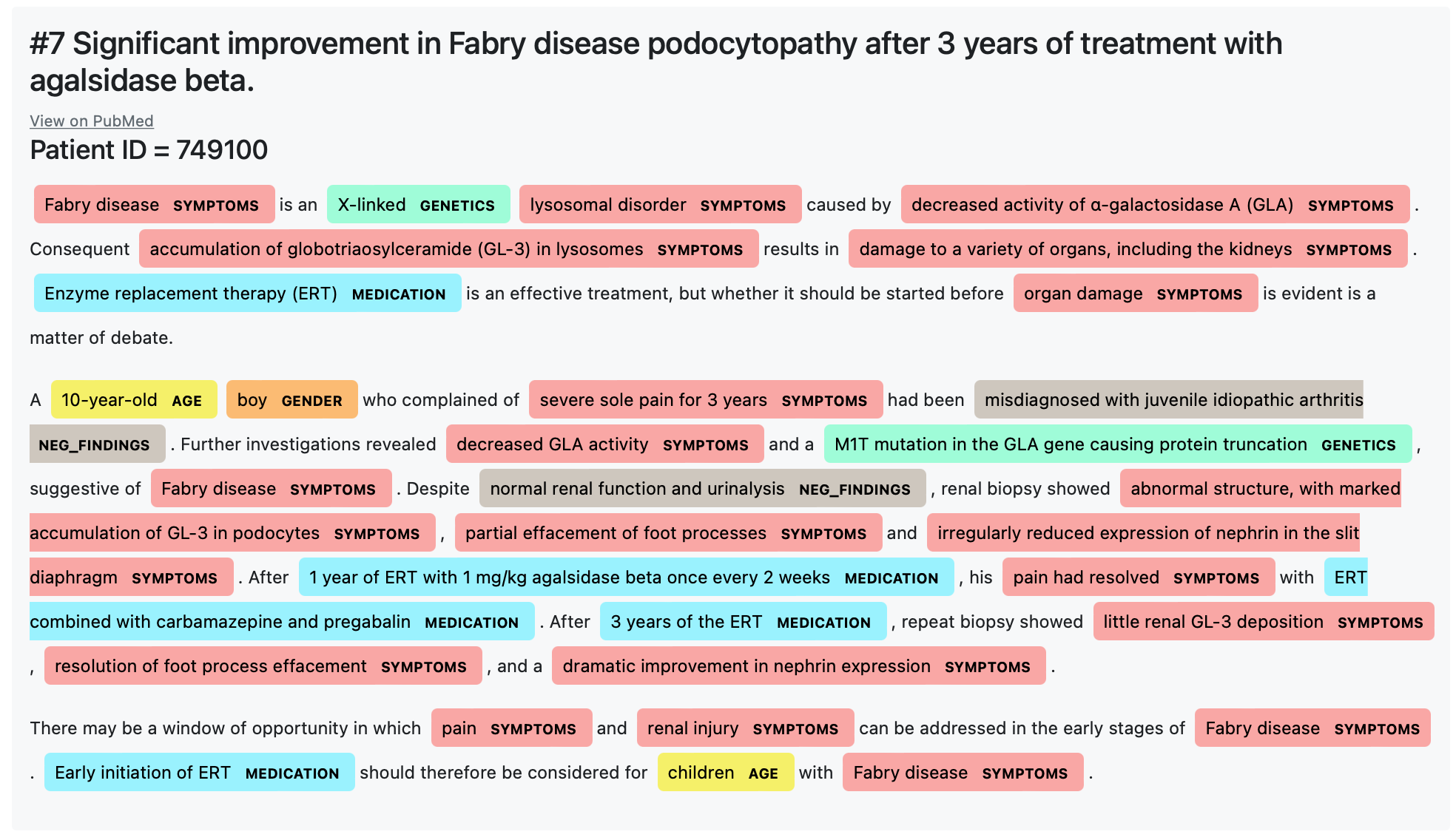


#### S3 Fig. COVID-19: “Case report: one case of coronavirus disease 2019 (COVID-19) in a patient co-infected by HIV with a normal CD4+ T cell count”


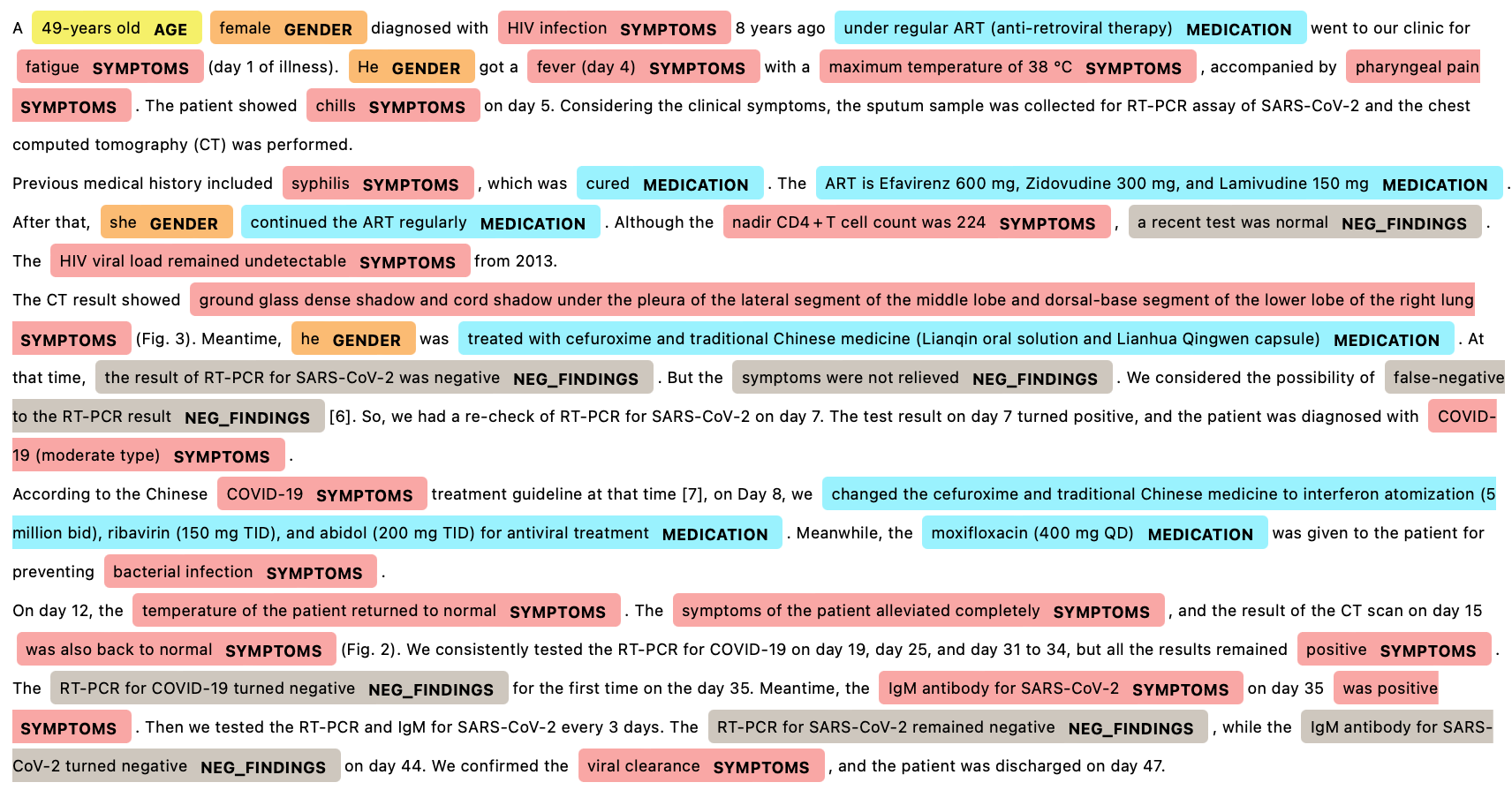


##

##

## Search Engine

**Solr configuration**

We have built the search engine on Apache Solr using the default similarity function Okapi BM25 (solr.apache.org version 8.11.1) [30]. Solr is configured to ignore the standard list of English stop words and to ignore capitalization. At query time, a predefined list of synonyms derived from the Unified Medical Language System (UMLS) is used to expand medical terms to also match their known synonyms [28].

**Disease-specific indexes**BM25 is built on TF-IDF, which follows the principle that rare terms are more informative, and therefore more relevant than frequent ones. Therefore, we built a separate index for the symptom field of each disease, so the ranking function can be scaled with regard to the frequency of occurrence of symptoms relative to each disease.

**Composite ranking function**We indexed each of the eight segmentation categories using a separate Solr field. One additional field was added to promote recent articles (published after 2005). The final ranking function was defined as a weighted sum of the field-wise BM25 scores. Furthermore, we penalised articles containing symptoms that were labelled as negative findings in the query. The weights for each field have been selected based on qualitative assessment and feedback from medical experts.

## Diagnosis using FindZebra.com

We queried the FindZebra API for all patients using the unsegmented generated queries and filtered out terms that are too indicative of the diagnosis such as “Angiokeratoma” for Fabry disease. For each patient, we recorded the ranks of the articles whose title contains the right diagnosis among the top hundred results. In S2 Table, we report the average recall, average precision at ten and twenty as well as the mean reciprocal rank up to rank hundred and the proportion of patients for which the correct diagnosis appears in the top ten and twenty.^14^ We found Gaucher disease to be much harder to diagnose, articles about Gaucher appeared in the first 20 results in only 31·1% of the patients, whereas this value was more than twice as high for the Fabry diagnosis (75·6%).

| S2 Table. Diagnosis using FindZebra.com: precision, recall, mean reciprocal rank (MRR) and proportion of patients for which the correct diagnosis appears in the retrieved documents. | | | | | | |
| --- | --- | --- | --- | --- | --- | --- |
|  | **Fabry** | | | **Gaucher** | | |
| **Group** | **all** | **atypical** | **typical** | **all** | **atypical** | **typical** |
| **Patients** | 4471 | 2491 | 1980 | 1095 | 698 | 397 |
| **Precision@10** | 19·3 | 24·2 | 13·3 | 3·5 | 4·1 | 2·4 |
| **Precision@20** | 11·5 | 14·3 | 8·1 | 3·0 | 3·3 | 2·3 |
| **Recall@10** | 32·2 | 40·3 | 22·1 | 4·1 | 2·3 | 1·3 |
| **Recall@20** | 38·5 | 47·5 | 27·1 | 3·3 | 3·7 | 2·6 |
| **MRR@100** | 0·53 | 0·66 | 0·35 | 0·09 | 0·11 | 0·06 |
| **Correct diagnosis in the top 10** | 68·4 | 82·3 | 51·0 | 21·7 | 23·8 | 18·1 |
| **Correct diagnosis in the top 20** | 75·6 | 88·1 | 59·9 | 31·1 | 34·0 | 26·2 |

## Evaluation

In this section, we describe the process followed to select patients for expert validation as well as the instructions that were given to the evaluators. Finally, we report measurements of the experts’ agreement.

### Selection of cases for expert validation

We aimed at selecting patients with rare phenotypes and diverse disease profiles. We used the diversity of the retrieved articles as a proxy for the diversity of the patients’ disease profiles. To select N=20 patients we

1. Started from the subset of patients with at least one atypical symptom (rare phenotypes).
2. Retrieved the top three articles for each patient in this subset.
3. Iteratively sampled patients until the N patients were selected. At each iteration, we took the subset of the remaining patients whose articles had been sampled the least and randomly selected one patient from this pool.

### Rating system

Two rare disease experts (AL and DE) evaluated the relevance of the search results given the 20 patients for each disease (Fabry and Gaucher). Each patient was presented to the evaluator both as tabular data (survey data) and as a comma-separated query (a screenshot of the evaluation interface is available in S1 Fig).

For each case, the experts were asked to evaluate the clinical relevance of the top three returned articles on a scale from one to five (the description of the scale, as presented to the evaluators, is available in the next sections). An additional text field was added after each rating, so medical experts could add details to support their decision. For each patient and each expert, we report the maximum grade given among the three articles, which is akin to evaluating the precision (@3). We further measured the agreement of the two experts based on each abstract.

### Patient Presentation

Each evaluation patient is introduced to the expert using the raw tabular data and the formatted query. A screenshot from the evaluation interface is showcased in S4 Fig.

| S4 Fig. Evaluation interface. Screenshot of the patient presentation. |
| --- |
| 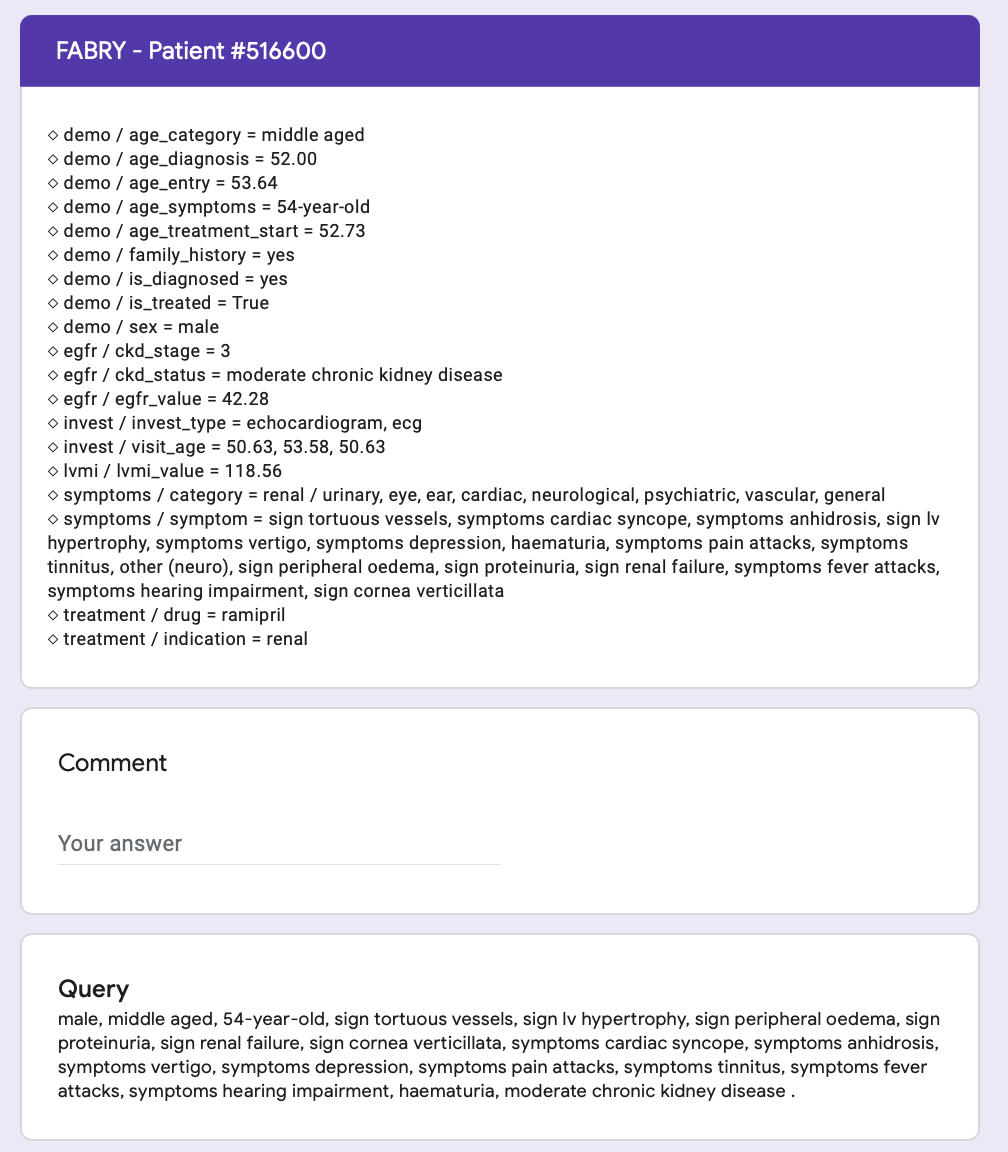 |

### Evaluation Scale

Experts were asked to grade the relevance of the retrieved articles using a one to five grading scale. In S3 Table, we present the description of the grading scale, as introduced to the experts. We further asked the experts how they interpreted the grading scale. The interpretation of the grading scale is presented in S4 Table (Expert 1 preferred commenting on each grade, whereas Expert 2 focused on the interpretation of the term “relevance”).

| S3 Table. Grading scale and task description | |
| --- | --- |
| **Task description** | “Your task is to evaluate how similar are the patients (query) and each of the medical cases presented in the PubMed abstracts. PubMed articles must be rated on a scale from 1 to 5 using the following scale:” |
| **1** | the article is totally irrelevant to the patient |
| **2** | the article doesn't reflect the main characteristics of the patient, although minor details are relevant |
| **3** | the article presents features that are relevant to the patient but important characteristics of the patients are not present in the article |
| **4** | the article discusses features that are relevant to the case, but other minor features are missing |
| **5** | the article matches very well the medical case, it would be very helpful to treat/diagnose the patient |

###

| S4 Table. Interpretation of the scale by each expert | | |
| --- | --- | --- |
| **Expert** | **Object** | **Comment** |
| **1** | **Grade 1** | All of the features discussed below [for grades 2-5] are lacking, and that some random buzzword was used as the basis of choosing the reference so that it would be of no clinical or strategic relevance to the case at hand |
| **1** | **Grade 2** | The article have very, very few or only one of the conditions that the patient at hand has, and as such there is probably no relevance to the patient at hand |
| **1** | **Grade 3** | The reference would need to have many of the bits of information I would like to see, but perhaps some novel or ancillary condition that might be relevant (but not necessarily). For example, in Gaucher, a newly described mutation or phenotype in the same ethnicity as the patient at hand even if the patient at hand could be milder or more severe than the one referenced; and for Fabry, for example, description of a new combination of therapeutic regimens in the gender or age range of the patient at hand even if all the symptoms and signs are not the same. |
| **1** | **Grade 4** | Closer to a "5" by having the same/very similar constellation of signs and symptoms as the patient at hand but the reader/clinicians would be able to make an educated guess as to whether it is really relevant to the patient at hand. |
| **1** | **Grade 5** | the reference would elucidate the case at hand so that it would be very helpful in informing my decision either clinically or strategically. This means that for Gaucher for example, it would have to have at minimum, confirmation of type, age of onset of symptomatic disease (i.e., not because of an incidental finding), and presence, type, and duration of Gaucher-specific therapy in addition to some (not just one) of the signs and symptoms that the patient at hand evinces. On the other hand, for Fabry, gender, age, and mutation are critical as well as presence, type, and duration of Fabry-specific therapy and symptomatic treatments (e.g., dialysis, cardiac meds, GI surgeries, and so on) in addition to some (not just one) of the signs and symptoms that the patient at hand evinces. |
| **2** | **Grades 1-5** | The grading scale - as presented - is sufficiently descriptive. |
| **2** | **Interpretation of the term**  **“relevance”** | The term relevance was interpreted as : “How good a match there is between case and abstract mostly considering actual presence of a given sign/symptom in the abstract that is also present in the case, but also - to a lesser extent - whether signs/symptoms noted in the abstract (and not found in the case) disturbs the interpretation or is wholly irrelevant.” |

### Experts’ agreement

In S5 Table, we measure the experts' agreement at the article level. We found that experts mostly disagreed on the relevance of the Fabry articles, 42% of the articles were given a similar score (tolerance ±1) whereas they mostly agreed on the Gaucher cases (87%). A closer inspection of the data showed that the experts had a higher agreement rate on the Gaucher cases that were rated one, confirming the irrelevance of the most returned abstracts. However, this trend was not identified in the rating of the Fabry articles.

| S5 Table. Experts’ agreement. Proportion of equal ratings for the two experts (60 articles per disease) | | | |
| --- | --- | --- | --- |
| **Disease** | **tolerance ±0** | **tolerance ±1** | **tolerance ±2** |
| **Fabry** | 15% | 42% | 67% |
| **Gaucher** | 72% | 87% | 92% |

## Case study

In this section, we study the case of patients F and G. We present the raw clinical data in S6 Table with expert comments in S7 Table. In S8 Table, we list the associated generated queries and the segmented versions in S9 Table. In S10 Table and S11 Table, we showcase the top three retrieved abstracts for each disease as well as their associated expert evaluation (grade and comment).

| S6 Table. Examples of survey data for Fabry and Gaucher patients | | | |
| --- | --- | --- | --- |
|  | **Fabry patient F** |  | **Gaucher patient G** |
| **Diagnosis** | Fabry | **Diagnosis** | Gaucher |
| **Age** | 66 | **Age** | 43 |
| **Sex** | male | **Sex** | male |
| **CKD stage** | 4 | **Haemoglobin** | 154 g/l |
| **eGFR** | 76·02 ml/min/1·73m^2^ | **Platelet count** | 87 10^9^/l |
| **LVMI** | 109·51 g/m**2·7 | **Liver size** | 4·60 multiples of normal |
| ·· | ·· | **Spleen size** | 0·85 multiples of normal |
| **Symptoms** | sign angiokeratomas, sign haemorrhoids, sign lv hypertrophy, symptoms vertigo, sign arrhythmia, haematuria, sign stroke, tumours, heart failure | **Symptoms** | lipid profile-low ldl, jaw-big osteolytic lesion, elevated ast, no hepatosplenomegaly |

| S7 Table. Expert Comments associated with each patient description | | | |
| --- | --- | --- | --- |
|  | **Fabry patient F** |  | **Gaucher patient G** |
| **Expert 1** | This is a 66 year old male patient with a family history who has started therapy currently. He has severe renal disease with hematuria, LVH with heart failure plus arrhythmias, stroke and "tumors" are noted. | **Expert 1** | This is a 43-year old male patient with no classic signs or symptoms of Gaucher disease other than thrombocytopenia which seems not to be caused by hypersplenism since there is no splenomegaly. It might be hypothesised that it is a result of the (untreated?) osteolysis which could have other causes. Whereas the jaw is a long bone that has been known to be affected by lesions (Gaucher cell infiltration) including a potential site of osteonecrosis, treatment of the lesion (low-dose, short-term steroids?) may resolve this without assuming it is Gaucher cell-induced. Elevated AST and overIt should be noted that Gaucher cell infiltration is putatively benign although it appears like lacunae in the jaw. liver enzymes and low LDLs are not Gaucher-related. |
| **Expert 2** | ·· | **Expert 2** | Why spleen-size 4,6 and at same time no splenomegaly? |

| S8 Table. Example of generated queries | | | |
| --- | --- | --- | --- |
|  | **Fabry patient F** |  | **Gaucher patient G** |
| **Generated query** | male, elderly, 66-year-old, sign angiokeratomas, sign haemorrhoids, sign lv hypertrophy, sign arrhythmia, sign stroke, symptoms vertigo, haematuria, tumours, heart failure, severe chronic kidney disease | **Generated query** | 43-year-old, male, adult, thrombocytopenia, lipid profile-low ldl, jaw-big osteolytic lesion, elevated ast, no splenomegaly, no hepatosplenomegaly, normal haemoglobin level, no hepatomegaly. |

| S9 Table. Example of segmented queries | | | |
| --- | --- | --- | --- |
|  | **Fabry patient F** |  | **Gaucher patient G** |
| **age** | 66 | **age** | 43 |
| **sex** | male | **sex** | male |
| **symptoms** | sign angiokeratomas, sign haemorrhoids, sign lv hypertrophy, sign arrhythmia, sign stroke, symptoms vertigo, haematuria, tumours, heart failure, severe chronic kidney disease | **symptoms** | lipid profile-low ldl, jaw-big osteolytic lesion, elevated ast |
| **Negative findings** | ·· | **Negative findings** | no splenomegaly, no hepatosplenomegaly, normal haemoglobin level, no hepatomegaly |

| S10 Table. Fabry - Evaluation of patient F | | |
| --- | --- | --- |
| **Query** | male, elderly, 66-year-old, sign angiokeratomas, sign haemorrhoids, sign lv hypertrophy, sign arrhythmia, sign stroke, symptoms vertigo, haematuria, tumours, heart failure, severe chronic kidney disease | |
| **Retrieved abstract #1 - “Phenotype and genotype characterization and twin association in patients with Anderson-Fabry cardiomyopathy.”** | | |
| 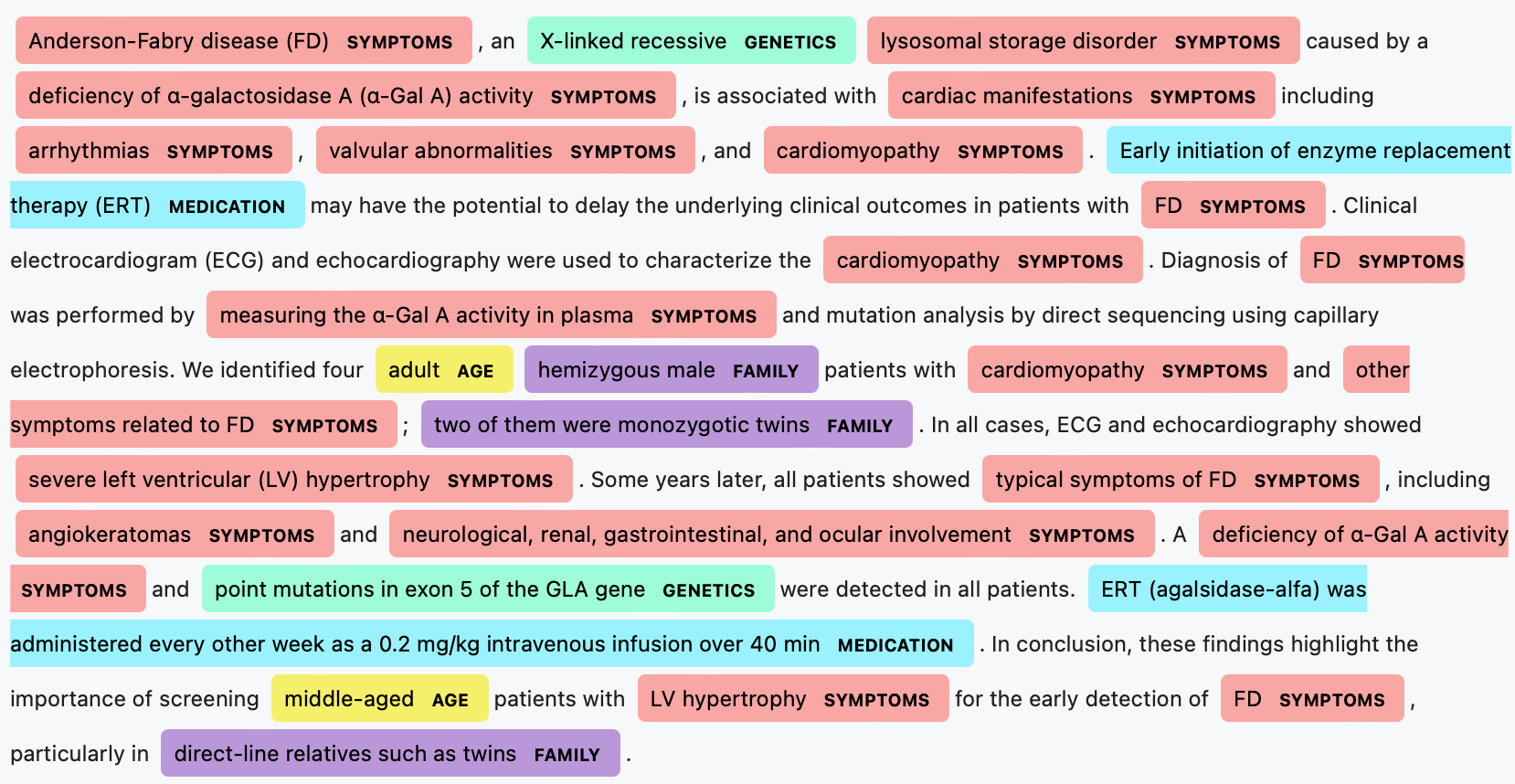 | | |
| **Review** | 1/5 | 5/5 |
| **Comment** | Since we do not know the genotypes of the case at hand, nor of those referenced in the report, this abstract is not helpful. | ·· |
| **Retrieved abstract #2 - “Radiofrequency ablation of ventricular tachycardia in Anderson-Fabry disease: a case series.**” | | |
| 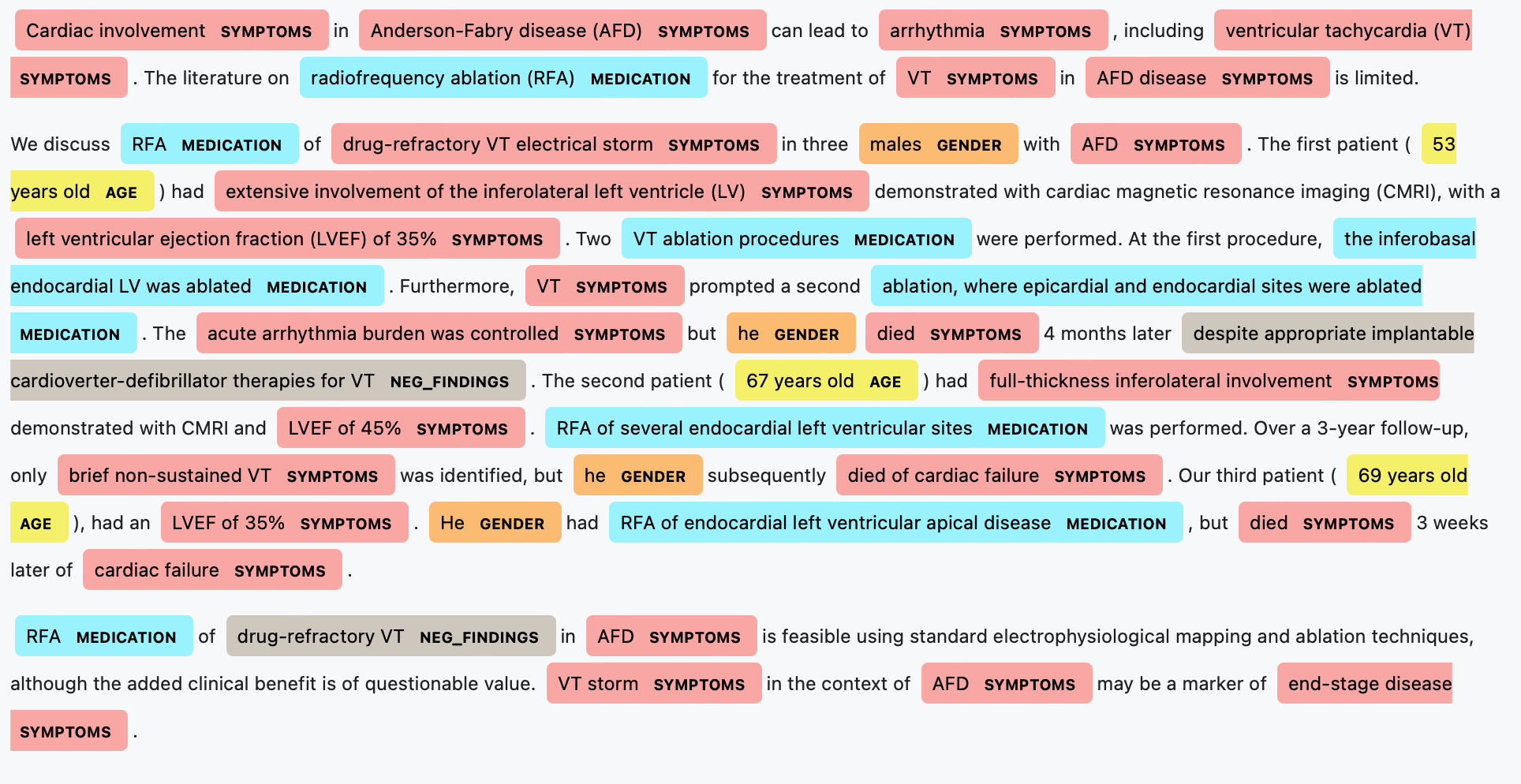 | | |
| **Review** | 1/5 | 2/5 |
| **Comment** | The histories of the patients in the reference do not speak well for the procedure (in advanced VT in Fabry), but since this is not one of the signs in the case at hand, it is irrelevant. | ·· |
| **Retrieved abstract #3 - “Successful Combined Heart and Kidney Transplantation in Patient With Fabry's Disease: A Case Report.”** | | |
| 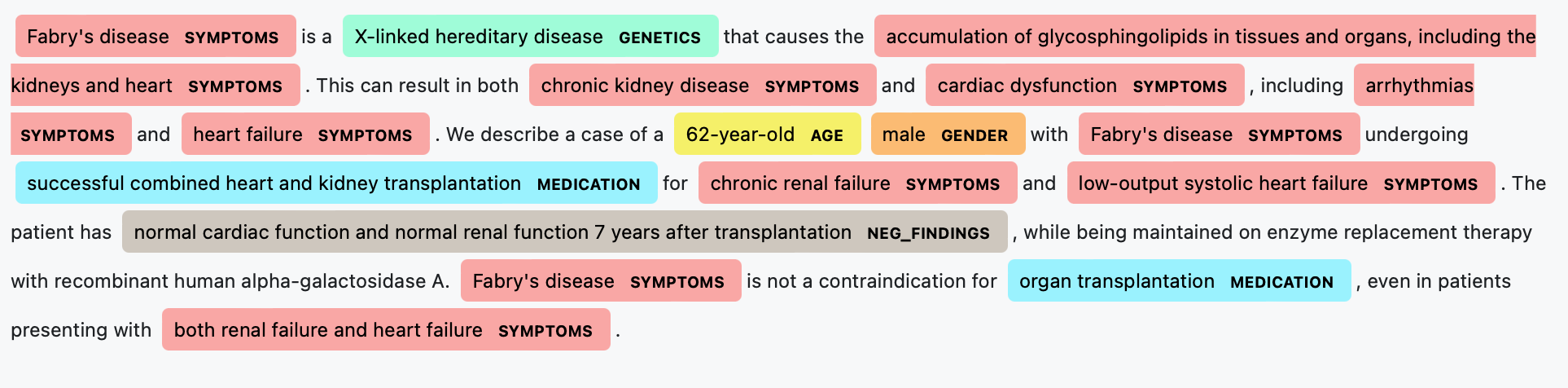 | | |
| **Review** | 3/5 | 3/5 |
| **Comment** | This reference is encouraging should the case at hand be a candidate for this complicated surgical intervention. | ·· |

| S11 Table. Gaucher - Evaluation of patient G | | |
| --- | --- | --- |
| **Query** | 43-year-old, male, adult, thrombocytopenia, lipid profile-low ldl, jaw-big osteolytic lesion, elevated ast, no splenomegaly, no hepatosplenomegaly, normal haemoglobin level, no hepatomegaly | |
| **Retrieved abstract #1 - “Bilateral symmetrical cortical osteolytic lesions in two patients with Gaucher disease.”** | | |
| 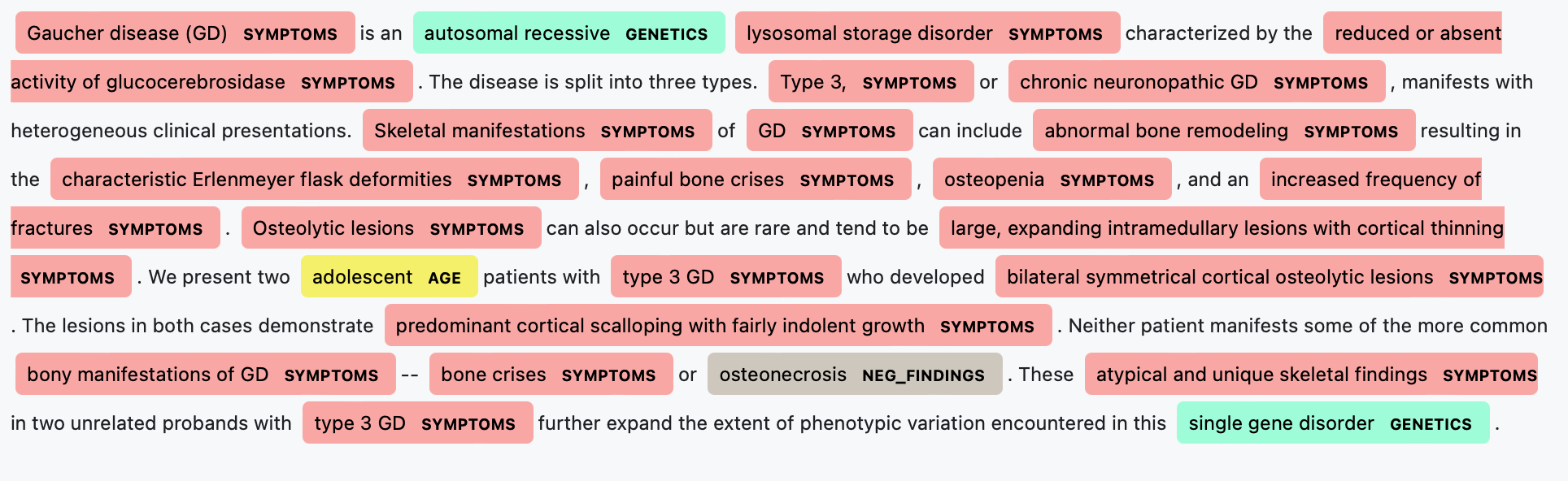 | | |
| **Review** | 2/5 | 2/5 |
| **Comment** | This reference underscores the fact that Gaucher cell infiltration which appears as a lesion that per force causes cortical thinning, need not necessarily have clinical ramifications...they look like lacunae and are not necessarily pain-inducing or necrosis-inducing. Yet, there is the slight comparability to the case at hand, despite the fact that the reference cases are type 3 where skeletal involvement (other than spinal curvature) and certainly one crisis are more rare than in type 1 patients, and in general bilateral bony lesions in Gaucher are rare. | Some relevance (osteolysis) but history of patient incompatible with GD3 |
| **Retrieved abstract #2 - “The nature and extent of jaw involvement in Gaucher disease: observations in a series of 28 patients.”** | | |
| 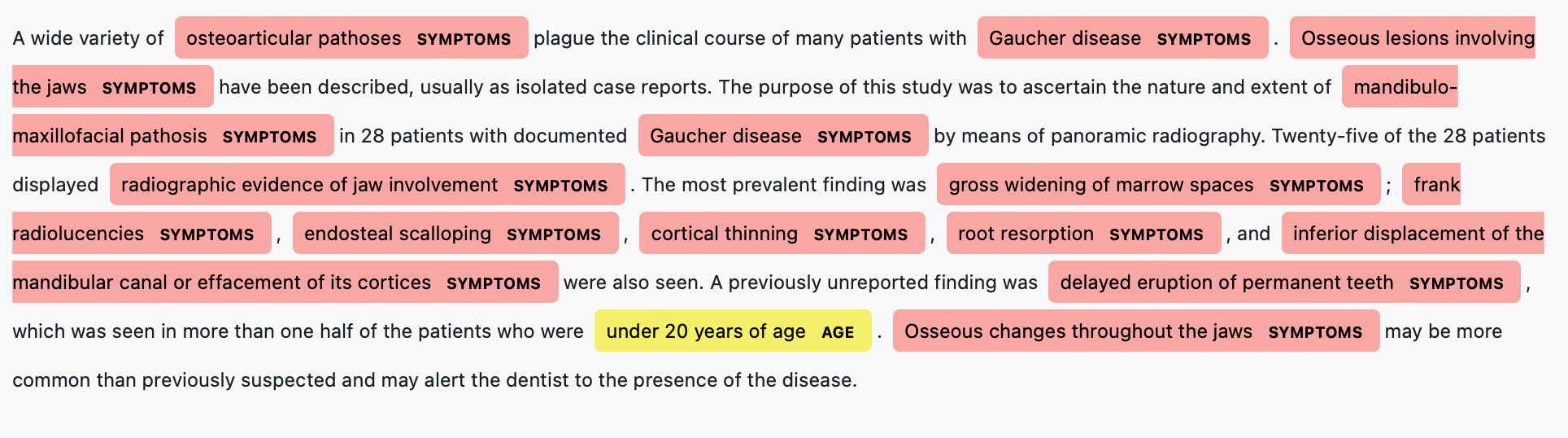 | | |
| **Review** | 5/5 | 1/5 |
| **Comment** | This reference indeed is the first and most definitive exploration of the involvement of the jaw in Gaucher (it is from my clinic). Nonetheless, while it is an excellent background for the jaw in Gaucher, it is not a case report but a survey. Nonetheless, having read this paper, a reader (like myself) would probably conclude that the jaw involvement in the case at hand, is not typical to Gaucher of the jaw and that the untreated osteolysis may explain the thrombocytopenia. | ·· |
| **Retrieved abstract #3 - “Bilateral Femoral Osteolytic Lesions in a Patient with Type 3 Gaucher Disease.”** | | |
| 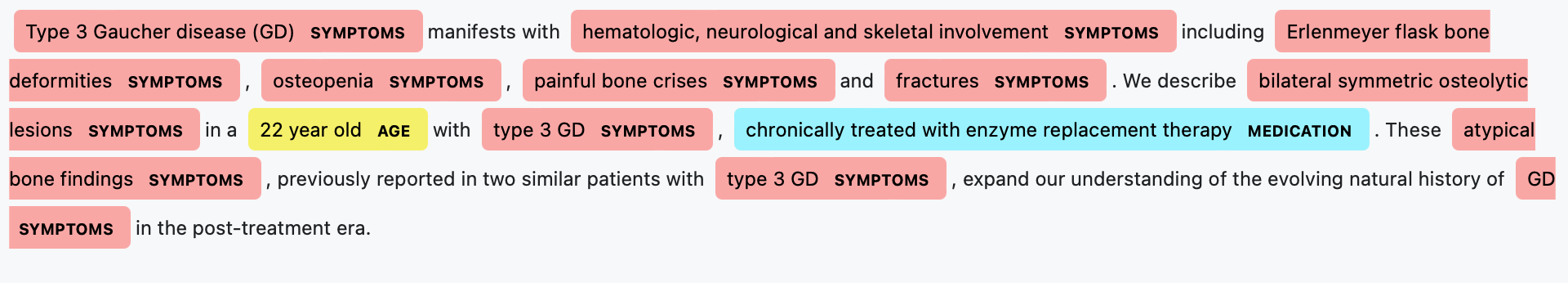 | | |
| **Review** | 2/5 | 2/5 |
| **Comment** | As in the original report of 2 unrelated type 3 patients, this report is rather non-informative about the case at hand because, although it reiterates the story of bilateral lesions (still apparently asymptomatic because there is no osteonecrosis), it now mentions enzyme therapy which might not be a first-line treatment for the case at hand that has no other Gaucher-specific signs. | ·· |

##

## Analysis of the clinical data

In this section, we provide further analysis of the clinical data (survey data) and the PubMed case reports.

### Demographics

In the S5 Fig, we present the age and sex distribution for the population of patients recorded in the PubMed case reports. In S6 Fig, we describe the population registered in the survey data.

| S5 Fig. PubMed case reports - patients demographics | |
| --- | --- |
| **Fabry** | **Gaucher** |
| 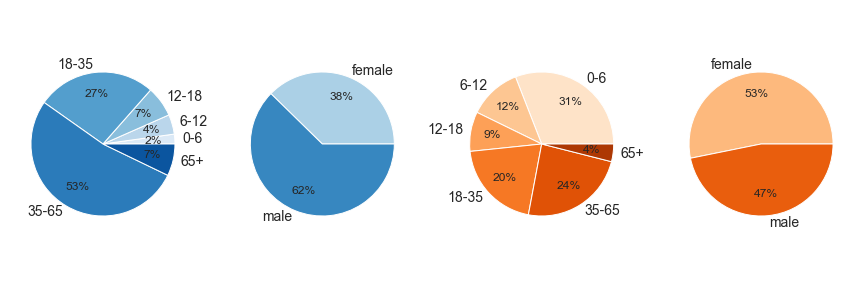 | |

| S6 Figure. Clinical data - patients demographics | | |
| --- | --- | --- |
| **Fabry** |  | **Gaucher** |
| 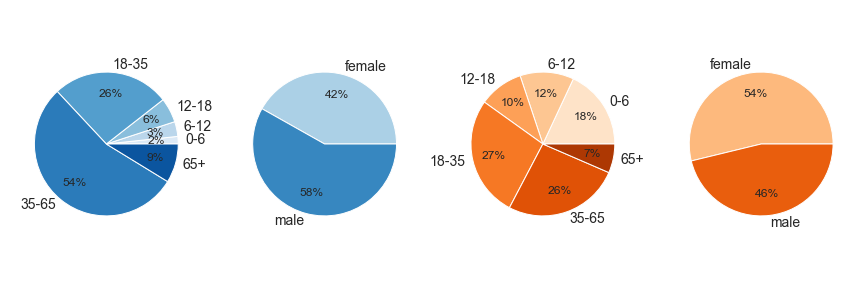 | | |

###

### Symptoms

In the S7 Fig (Fabry) and S8 Fig (Gaucher), we report the frequency of symptoms as recorded in the survey data on the left side of the plots. We used a different colour for symptoms considered as typical or atypical. We found the distribution of the Gaucher symptoms to have a longer tail than the distribution of the Fabry symptoms.

On the right side of the plots, we report the frequency of symptoms occurring in the PubMed case reports. Because extracted segments are heterogeneous, we extracted symptoms from the PubMed abstracts using the segments labelled as “symptoms” using our tool, and further extracted UMLS entities using the ScispaCy library (we only accepted TUIs within the list T019, T020, T033, T037, T046, T047, T184, T190, and T191). We counted only one occurrence of symptoms per article. The frequencies of PubMed symptoms are under-estimated due to imperfect entity matching (for instance, we found both “kidney diseases”, “kidney failure” and “chronic kidney disease stage 5” in the distribution of Fabry symptoms, S7 Fig).

| S7 Fig. Fabry. Distribution of the top 80 symptoms in the survey data and in the PubMed case reports |
| --- |
| 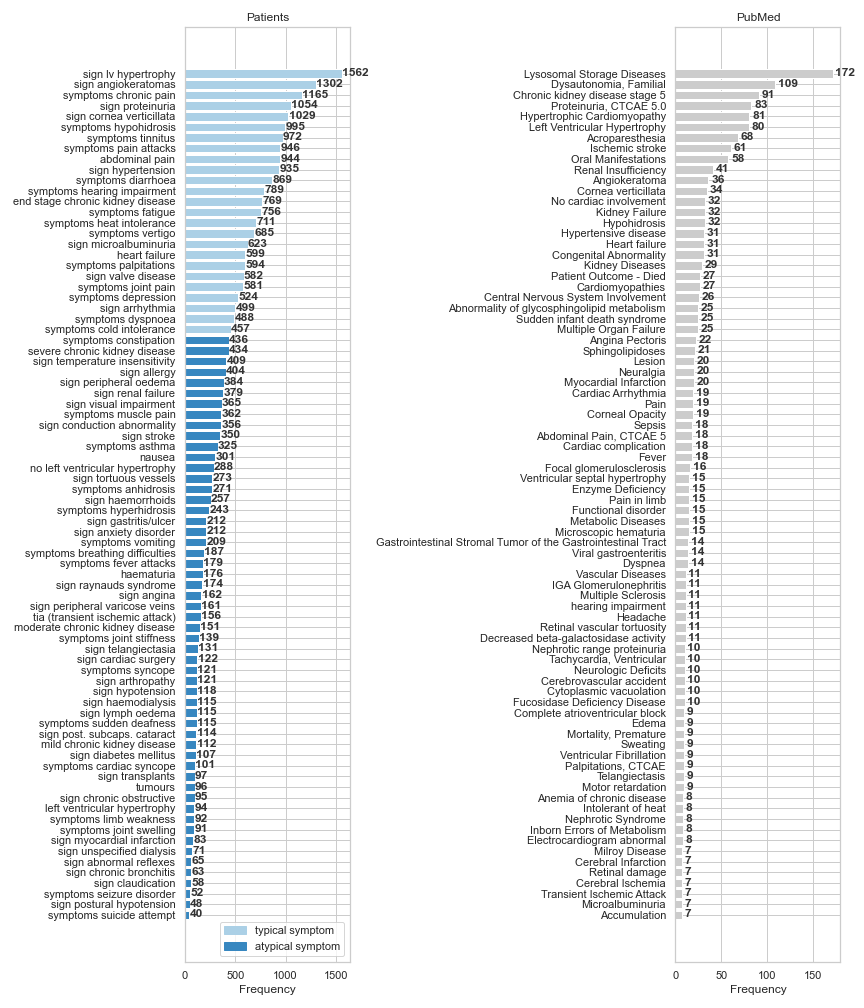 |
| S8 Fig. Gaucher. Distribution of top-80 symptoms in the survey data and in the PubMed case reports |
| 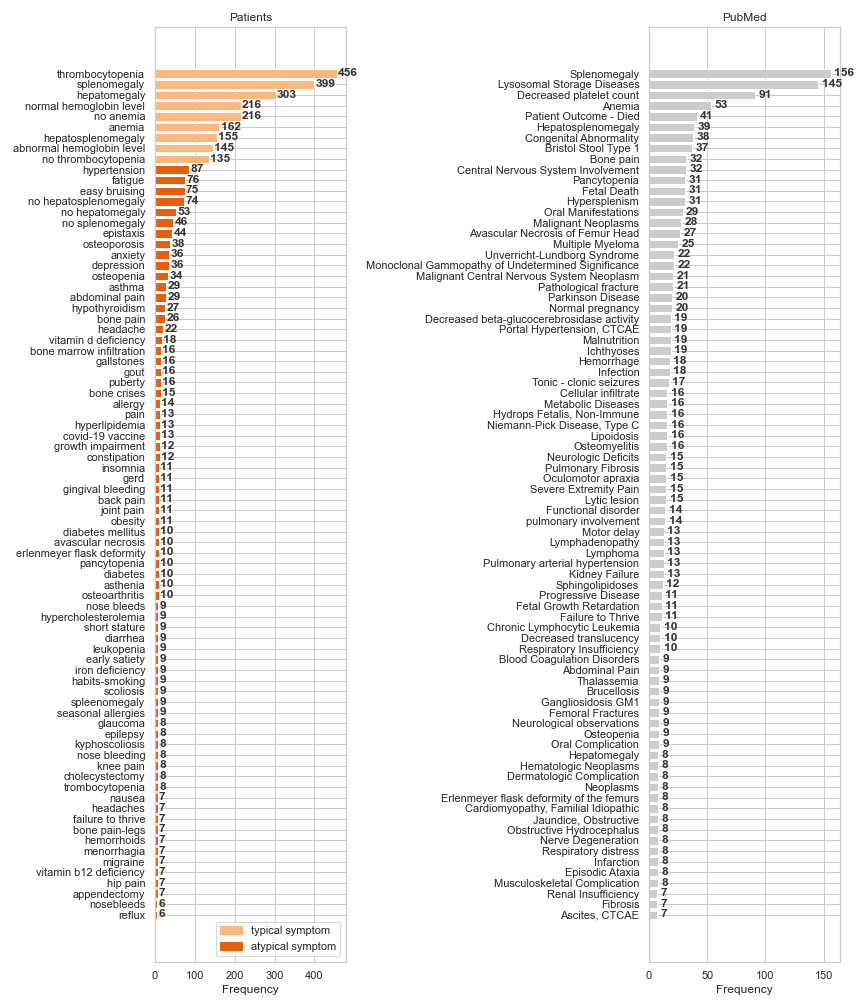 |

### Population and Corpora

In S9 Fig and S10 Fig, we present a visualisation of the patient-article network for the Fabry and Gaucher patients. We identified clusters of patients and articles using the Louvain community detection algorithm using the same parameters for each disease. We identified 8 clusters for Fabry, and 13 for Gaucher. We summarised the features for the top four communities in S12 Table and S13 Table (age, sex, proportion of patients following treatment, and the most relevant symptoms). Symptom relevance is evaluated using TF-IDF across clusters of patients, only symptoms occurring in more than 5 patients are reported.

For both diseases, the clusters were characterised by different populations, as we recorded significant differences in their distributions of age, sex, and treatment. These characteristics are further supported by the list of relevant symptoms, which suggest different disease profiles.

The structures of the networks highlight exhibit differences that support the results presented in S12 Table. Whereas the Fabry network displays a centralised structure, with a few nodes dominating the network, the Gaucher network appears more uniform, with a more distributed structure. The centralised structure of Fabry is also implied by the number (8) and the non-uniform sizes of its top-four clusters (162, 122, 62 and 53 patients), whereas clusters are more uniform (77, 78, 57, and 55 patients) and more numerous (13) in Gaucher.

The analysis of the structure allows us to draw a different picture for each disease. Fabry is described by a small number of dominant profiles, whereas Gaucher profiles appear to be more diverse.

| S9 Figure. 500 Fabry patients and their top-three retrieved articles. Groups of patients and articles are identified using automatic tools. S12 Table summarises the patients’ characteristics for each group. |
| --- |
| 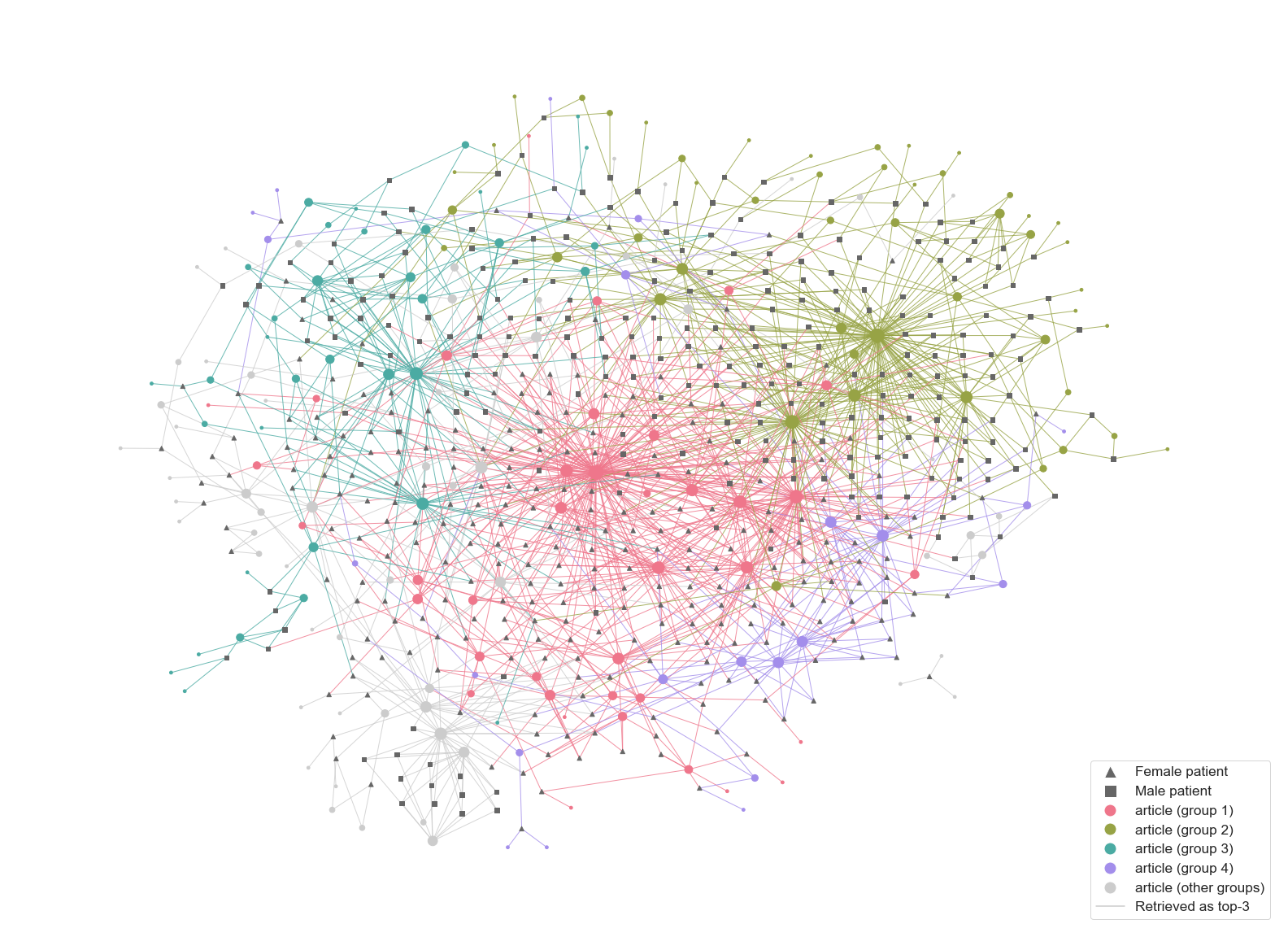 |

| S12 Table. Summary of the Fabry patients found in the top four clusters extracted from the patient-article network (S6 Fig) | | | | |
| --- | --- | --- | --- | --- |
| **Group** | **1** | **2** | **3** | **4** |
| **Patients** | 162 | 122 | 62 | 53 |
| **Articles** | 38 | 49 | 35 | 22 |
| **Age** | 49 ± 22 | 38 ± 19 | 62 ± 14 | 38 ± 22 |
| **Sex** | 77% females | 98% males | 68% males | 85% females |
| **Treated** | 61% | 80% | 65% | 58% |
| **Top Symptoms (TF-IDF)** | other (genital)  arthropathy  chronic bronchitis  chronic obstructive  angina  joint stiffness  breathing difficulties  myocardial infarction  peripheral varicose veins  cardiac syncope  hyperhidrosis  tia (transient ischemic attack)  abnormal reflexes  other (vascular)  cardiac surgery  postural hypotension  tumours  joint swelling  limb weakness  hypotension  other sign or symptom  haemorrhoids  lymp oedema  sudden deafness  gastritis ulcer  transplants  other (ear)  syncope  anxiety disorder  diabetes mellitus | suicide attempt  unspecified dialysis  lymph oedema  transplants  haemodialysis  telangiectasia  breathing difficulties  joint swelling  other (ear)  myocardial infarction  post subcaps cataract  syncope  limb weakness  cardiac surgery  other infections  chronic obstructive  fever attacks  cardiac syncope  sudden deafness  anxiety disorder  other (genital)  muscle pain  raynauds syndrome  vomiting  joint stiffness  hypotension  peripheral varicose veins  other (respiratory)  angina  haematuria | diabetes mellitus  unspecified dialysis  cardiac syncope  haemodialysis  gastritis ulcer  other (endocrine)  haemorrhoids  haematuria  allergy  other (eye)  stroke  other (musculoskeletal)  renal failure  other (dermatological)  conduction abnormality  other (renal urinary)  valve disease  dyspnoea  palpitations  microalbuminuria  other (gastrointestinal)  heart failure  arrhythmia  fatigue  diarrhoea  vertigo  other (neuro)  hypohidrosis  hearing impairment  chronic pain | hypotension  vomiting  abnormal reflexes  other infections  syncope  limb weakness  nausea  gastritis ulcer  fever attacks  peripheral varicose veins  other sign or symptom  muscle pain  other (endocrine)  raynauds syndrome  other (respiratory)  allergy  asthma  visual impairment  tortuous vessels  other (eye)  constipation  temperature insensitivity  other (musculoskeletal)  cold intolerance  other (dermatological)  peripheral oedema  conduction abnormality  stroke  renal failure  valve disease |

| S10 Figure. 500 Gaucher patients and their top-three retrieved articles. Groups of patients and articles are identified using automatic tools. S13 Table below summarises the patients’ characteristics for each group. |
| --- |
| 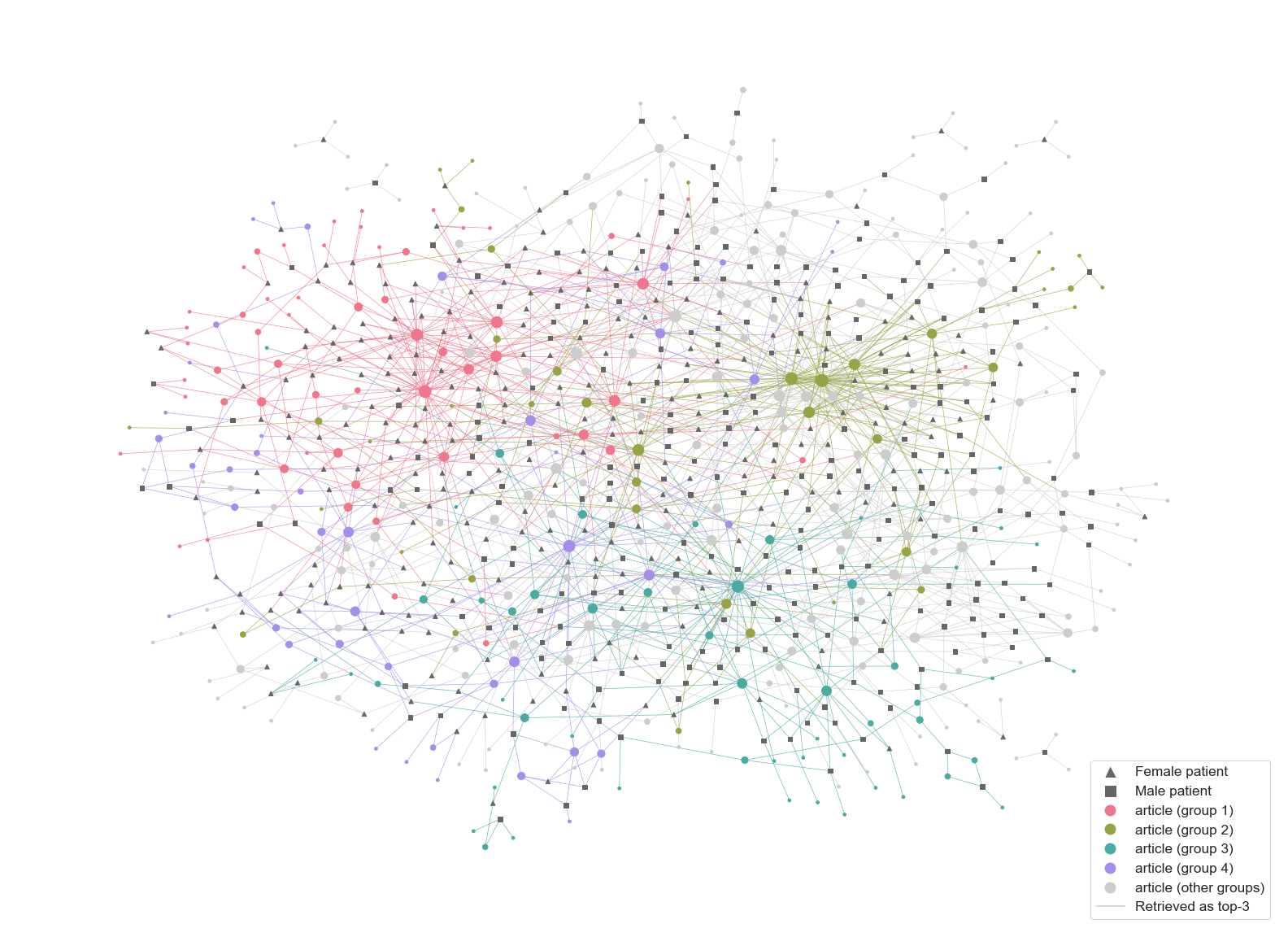 |

##

| S13 Table. Summary of the Gaucher patients found in the top four cluster extracted from the patient-article network (S7 Fig) | | | | |
| --- | --- | --- | --- | --- |
| **Group** | **1** | **2** | **3** | **4** |
| **Patients** | 77 | 78 | 57 | 55 |
| **Articles** | 57 | 41 | 52 | 43 |
| **Age** | 22 ± 28 | 18 ± 26 | 34 ± 42 | 37 ± 42 |
| **Sex** | 90% females | 68% males | 58% males | 65% females |
| **Treated** | 79% | 85% | 81% | 85% |
| **Top Symptoms (TF-IDF)** | epistaxis  hypothyroidism  hepatomegaly  hepatosplenomegaly  easy bruising  osteoporosis  anaemia  splenomegaly  hypertension  thrombocytopenia | erlenmeyer flask deformity  bone marrow infiltration  osteopenia  fatigue  bone crises  anxiety  easy bruising  abdominal pain  hepatomegaly  anaemia  thrombocytopenia  splenomegaly  hypertension | headache  allergy  easy bruising  hepatomegaly  anaemia  hypertension  splenomegaly  thrombocytopenia | pulmonary hypertension  depression  anxiety  hypertension  anaemia  thrombocytopenia  splenomegaly |
