# Supplementary material for: FindZebra online search delving into rare disease case reports using natural language processing
Source: PLOS Digit Health. 2023 Jun 29;2(6):e0000269. doi: 10.1371/journal.pdig.0000269 (PMC10309602; doi:10.1371/journal.pdig.0000269)
Supplement: Attachment — Submitted filename: Response to reviewers.pdf [file pdig.0000269.s003.pdf]

Reviewer #1 detailed written comments

Answer: We would like to thank Reviewer 1 for careful and constructive comments that we believe have helped to substantially improve the presentation in the paper.

MAJOR ISSUES
(1) Focus of the article should be sharpened: Should the study describe the evaluation process or how the tool works, or both?
Answer: Good point. It is actually both. We have updated the Author Summary and introductory text to make it more clear what our intended contributions are.

(2) Introduction:
(2a) The combined use of already existing models should be added when mentioning the two components for the tool (p. 3). Otherwise, the impression is created that the components were completely newly developed by the authors.
Answer: Good point. We have clarified what are existing tools and what are new ones.
(2b) The goal of the study is stated as the evaluation of the tool (p. 3). However, the title and the methods also describe the functional area of the extension. Therefore, the research objective (and possibly also the title) should be modified accordingly.
Answer: Good point. This has been clarified along the lines under point (1). We are open to changing the title. Suggestions are welcome.

(3) Material and methods:
(3a) When explaining the workflow, only the goal is named and not the method of functioning. A more detailed explanation – especially of Figure 2 – is necessary here (p. 3)
Answer: good point. Done.
(4) Results:
(4a) The description for "Search index" should focus more on actual results and less on the procedure (p. 7).
Answer: Good point. We have moved this subsection to Search engine subsection in Methods.
(4b) It is not always clear which results were obtained by which methods; especially with respect to the validation process, it would be interesting to know which results resulted from which step. Here, a reference to the individual steps would be important. (An example of this is the evaluation of the previous FindZebra tool (p. 8)).
Answer: We have clarified this.

MINOR ISSUES
(5) Introduction:
(5a) Transition from explanations of diseases to explanation of PubMed could seem more natural by adding the research gap again (p. 2).
Answer: Yes agreed. We have augmented the text here.
(5b) Claim that a tool is missing is not substantiated, so perhaps add "to our knowledge" (p. 3).
Answer: Added.
(5c) Incomplete sentence: “Case reports could be used to improve the clinical management of today’s patients, for instance by tailoring the treatment to the patient profile, or by supporting the healthcare providers in their [16,17]” (p. 3).

Answer: Updated.
(5d) Figure 1 is referenced when the two components are mentioned, but the two components are not explicitly shown in the figure. Here, you could highlight the individual components separately (p. 3).
Answer: Good point. Updated.

(6) Material and Methods:
(6a) The introduction to the chapter "Material and methods" does not exactly match the following subchapters. The title and sequences should be harmonized here (p. 3).
Answer: Good point. Fixed.
(6b) Is this really de facto anonymization? Or can individual patients be identified by the combination of sex, age and mutation (p. 4)?
Answer: Neither Fabry or Gaucher or the mentioned mutations are so rare that there is any significant risk of deanonymization.
(6c) Explanation of text segmentation should be under the heading "Text Segmentation" and not be content of "Data" (p. 5).
Answer: Updated.

(7) Results:
(7a) The introduction to the chapter "Results" does not exactly match the following subchapters. The title and sequences should be harmonized here (p. 7).
Answer: Good point. Fixed.
(7b) Revision of figures and tables necessary:
(7b.i) Figure 4: Name / describe units (p. 10).
(7b.ii) Labeling of table 7 differs from other labeling (p. 9).
(7b.iii) No table 6 included (p. 8-9).
(7b.iv) Two figures are named “Figure 4” (p. 9 and 11).
Answer: Fixed.

(8) Conclusion:
(8a) Improve the conclusion by describing the novelty of the approach in more detail and by stating scientific and technical implications for the entire research field (p. 13).
Answer: Good point updated.

OTHER POINTS
(9) Great unified and beautiful presentation of illustrations.
Answer: Thank you!
(10) Great detailed discussion of the results.
Answer: Thank you!

Reviewer #2

Reviewer #2: The manuscript describes a new search functionality for rare disease case reports of FindZebra.com service. Two rare diseases of Gaucher and Fabry were used as the exemplar study diseases. Natural language processing models/tools were utilised to support the modelling/indexing of case reports and the matching between user queries and case reports. Particularly, user queries were

generated from 'real' patient cases including age/gender and clinical features like symptoms and phenotypes. Evaluation protocols and metrics were proposed to validate the utilities of the service in supporting clinical decision making for patients with those diseases, seemingly in scenarios of both diagnosis and treatments.

Overall, from clinical utility point of view, this would potentially be a very valuable work and much needed service for supporting rare disease diagnosis, treatment and managements. However, technically - from information retrieval and NLP point of view, the work requires further developments and clarifications to make it publishable - in other words, making substantial contribution to the field and useful for the community.

1. It is not clear how NLP models were used and developed. Named entity recognition was mentioned only in the abstract. In the main text and supplementary it was called segmentations. The two might be totally different NLP tasks. The use of terminology aside, there is no information how the NER was done. PubmedBERT was mentioned to be the language model for fine-tuning the segmentation task (assuming the NER for clinical features like symptoms etc). However, there was no mention where the ground truth of NER came from.
Answer: Good point. We have substantially updated the description of the solution so that the terminology is unified and it becomes more clear how we labeled the data and use the BERT model. In particular, we removed mentions of the NER task as we only explored the task of text segmentation. Note that we aimed at labeling longer chunks of text than being done for the NER tasks. The main motivation was to automatically extract a structured patient profile.

2. The ranking algorithm is key in the methodology of an information retrieval system. But it seems very limited contribution was proposed to that aspect - the default BM25 was used instead.
Answer: It is not true that we use BM25 only. BM25 is applied to each after categorization of terms in the NER step. This has been clarified in the updated manuscript. Thank you for pointing this out.

3. The key evaluation result of precision@3 seems not very good. There were 5 grades as detailed in the supplementary. From the descriptions, only grade 4 & 5 can be assessed to be relevant. From table 7, the 'real' useful results for the two diseases were 52% and 8% for the two diseases respectively. This seems a bit low for a search service according to these numbers. There was no baseline provided to compare their service to. Also, there was no ablation evaluation on different techniques. Therefore, it is not clear how difficult the task was and it is hard to justify how good their service was.
Answer: It is true that the evaluation results are not strikingly good. However, this is a very difficult task to find a case story exactly matching a given patient's case as the cases were selected to be non-generic and thus hard. The low score for Gaucher identified the importance of using up to date literature (due the changing characteristics of the disease following new treatment options). We agree that reporting baseline results would make this work better. Nevertheless, we preferred to allocate all resources to evaluate more patient cases (40 patients, 1 method) rather than evaluating fewer patients (20 patients, 2 methods). We collaborated with the medical experts to provide an in-depth qualitative review of the tool in the discussion section. We hope that quantitative, qualitative and the release of the evaluation, and the tool being open-source will provide the public with sufficient data. We have clarified the article text to address this point.

4. From table 8 (analysis of the failure patterns), first, clearly the domain experts were evaluating the service from facilitating diagnosis/treatment points of view. For example, item 2 was "The article is outdated", which is clearly not a direct IR/NLP problem. However, the method/models proposed by the

authors seem not dealing with these requirements directly. This leads to a general question - have the authors properly defined their technical tasks based on the intended use of the service?
Answer: Clearly, the proposed method has been designed from the perspective of supporting medical domain experts. The outdated issue of the Gaucher literature was something that we became aware of in the preliminary analysis and spurred updates to the evaluation methodology and the final online tool (by introducing a publication selector).

5. A side and puzzled finding from table 8 was the big difference of the last two rows between the two diseases. There seems to be no obvious reasons why the second disease was harder for the same (relatively simpler) IR tasks.
Answer: Good point. See answer to point 3.

6. I struggled to understand the relevance of "Population and Corpora" section. First, it seems not directly relevant to information retrieval and NLP tasks. Second, the networks/graphs were generated from top 3 articles of the service. However, the performance of the current service as it is seems not good enough to support the generation of a reasonably good network for this kind of analysis.
Answer: The network analysis aims at illustrating how the search function maps the populations of patients to the PubMed corpora. We updated the manuscript to make our intent more clear. Although our retrieval function is imperfect, the retrieved top 3 abstracts share lexical overlap with the patient description (BM25). The motivation for this experiment was to check that the population of patients is distributed across numerous articles, whch we summarized in the text with the average number of patients per article. Nevertheless, we hope that the network visualization and the cluster analysis in the Supplement will help illustrate our work better.

Presentation issues:
- There are no justifications on why the two rare diseases. Do they present two exemplar (distinct) IR/NLP challenges?
- The abstract does not provide clear descriptions on what the main validation purposes are.
- There are many abbreviations which should be expanded when they were first used.
- There are grammar and incomplete sentences at various places in the manuscript.

Answer: Good points similar to those of Reviewer 1. We have updated primarily the Abstract and Introduction to make these points more clear. Thank you for helping us improve the presentation and the paper.
